# Pathogenic Variant Frequencies in Hereditary Haemorrhagic Telangiectasia Support Clinical Evidence of Protection from Myocardial Infarction

**DOI:** 10.3390/jcm13010250

**Published:** 2023-12-31

**Authors:** Kinshuk Jain, Sarah C. McCarley, Ghazel Mukhtar, Anna Ferlin, Andrew Fleming, Deborah J. Morris-Rosendahl, Claire L. Shovlin

**Affiliations:** 1 National Heart and Lung Institute, Imperial College London, London W12 0NN, UK; kinshuk.jain21@imperial.ac.uk (K.J.); sarah.mccarley22@imperial.ac.uk (S.C.M.); ghazel.mukhtar18@imperial.ac.uk (G.M.); d.morris-rosendahl@rbht.nhs.uk (D.J.M.-R.); 2 Clinical Genetics and Genomics Laboratory, Royal Brompton Hospital, Guy’s and St Thomas’ NHS Trust, London SE1 7EH, UK; a.ferlin@rbht.nhs.uk (A.F.); a.fleming@rbht.nhs.uk (A.F.); 3 Specialist Medicine, Hammersmith Hospital, Imperial College Healthcare NHS Trust, London W12 0HS, UK; 4 Social, Genetic and Environmental Determinants of Health, NIHR Imperial Biomedical Research Centre, London W2 1NY, UK

**Keywords:** atherosclerosis, dominant negative activity, heart attack, low-density lipoprotein (LDL) transcytosis, missense variant

## Abstract

Hereditary haemorrhagic telangiectasia (HHT) is a vascular dysplasia inherited as an autosomal dominant trait, due to a single heterozygous loss-of-function variant, usually in *ACVRL1* (encoding activin receptor-like kinase 1 [ALK1]), *ENG* (encoding endoglin [CD105]), or *SMAD4*. In a consecutive single-centre series of 37 positive clinical genetic tests performed in 2021–2023, a skewed distribution pattern was noted, with 30 of 32 variants reported only once, but *ACVRL1* c.1231C>T (p.Arg411Trp) identified as the disease-causal gene in five different HHT families. In the same centre’s non-overlapping 1992–2020 series where 110/134 (82.1%) HHT-causal variants were reported only once, *ACVRL1* c.1231C>T (p.Arg411Trp) was identified in nine further families. In a 14-country, four-continent HHT Mutation Database where 181/250 (72.4%) HHT-causal variants were reported only once, *ACVRL1* c.1231C>T (p.Arg411Trp) was reported by 12 different laboratories, the adjacent *ACVRL1* c.1232G>A (p.Arg411Gln) by 14, and *ACVRL1* c.1120C>T (p.Arg374Trp) by 18. Unlike the majority of HHT-causal *ACVRL1* variants, these encode ALK1 protein that reaches the endothelial cell surface but fails to signal. Six variants of this type were present in the three series and were reported 6.8–25.5 (mean 8.9) times more frequently than the other *ACVRL1* missense variants (all *p*-values < 0.0039). Noting lower rates of myocardial infarction reported in HHT, we explore potential mechanisms, including a selective paradigm relevant to ALK1′s role in the initiating event of atherosclerosis, where a plausible dominant negative effect of these specific variants can be proposed. In conclusion, there is an ~9-fold excess of kinase-inactive, cell surface-expressed *ACVRL1*/ALK1 pathogenic missense variants in HHT. The findings support further examination of differential clinical and cellular phenotypes by HHT causal gene molecular subtypes.

## 1. Introduction

Hereditary haemorrhagic telangiectasia (HHT) is a vascular dysplasia inherited as an autosomal dominant trait. First described as a familial nosebleed/anaemia condition with what are now termed mucocutaneous telangiectasia [1,2,3], it was renamed Osler–Weber–Rendu syndrome following further reports [4,5,6]. With time, it was recognised that some family members were affected by gastrointestinal telangiectasia [7] and/or internal arteriovenous malformations (AVMs), particularly in the lungs [8,9,10], liver [11], and brain [12,13,14]. Following international consensus in 1997, these elements were formalised into the Curaçao Clinical Diagnostic Criteria, which remain in current use [15]. The most common clinical manifestation is nose bleeds, and bleeding may also occur from the gastrointestinal tract [16]. Recurrent iron losses cause anaemia requiring iron supplementation and, in some cases, red cell transfusion. The nosebleeds may be severe, but they do not necessarily get worse with age (though, with time, more people have their first HHT nosebleed [17,18]), and a fluctuant pattern is observed [19]. In contrast, mucocutaneous and gastrointestinal telangiectasia do increase with age [17]. Most HHT-affected individuals have visceral AVMs, with pulmonary and hepatic being the most common (each affecting about 50% of patients), cerebral affecting fewer than 1 in 10 patients, and rarer AVMs in other viscera. Pulmonary AVMs are usually present by the end of puberty [20], with the age at development less clear for cerebral and hepatic AVMs [19]. There can be diverse medical complications due to HHT, for example, from anaemia and systemic AVMs that increase cardiac output; from paradoxical emboli through pulmonary AVMs that lead to ischaemic stroke and brain abscess; and, in a smaller number of patients, due to haemorrhage from cerebral vascular malformations. The interplay of the complex pathophysiology was recently articulated by the European Reference Network for Rare Vascular Diseases [21], and consensus guidance is available to guide clinical management [20,21,22,23,24,25].

Four different genes harbour pathogenic HHT-causal variants. For *ENG* (encoding endoglin (CD105)) and *ACVRL1* (encoding activin receptor-like kinase 1 (ALK1)), pathogenic variants were identified after linkage analyses in different families had localised the gene positions to intervals on chromosomes 9 and 12, respectively [26,27]. Both endoglin and ALK1 were known to mediate signalling by the transforming growth factor (TGF)-β superfamily [28]. At the same time, downstream signalling mediators of TGF-β signalling were being identified in Caenorhabditis elegans (*Sma* proteins) [29] and Drosophila melanogaster (Mad proteins, for ‘mothers against decapentaplegic’) [30], leading to the composite nomenclature of ‘SMAD’ [31]. *SMAD4* was identified as an HHT-causal gene following recognition of a phenotypic overlap with juvenile polyposis [32]. More recently, a fourth gene has been confirmed, as *GDF2*. This encodes the ALK1 ligand bone morphogenetic protein (BMP)9, which binds ALK1 with high affinity in dimeric form [33,34], after endoglin has first captured circulating BMP9 and ‘presented’ to ALK1 [35]. *GDF2* heterozygous variants were initially described as causing an HHT-like syndrome [36] and, more recently, in a family meeting full Curaçao Criteria clinical designation [37]. Genetic testing for *ACVRL1*, *ENG*, *SMAD4,* and *GDF2* is widely performed as part of HHT diagnostics [21,22,38,39,40,41,42,43,44,45]. Genotyped patients and families are commonly referred to as HHT type 1 (*ENG*, OMIM #187300), HHT type 2 (*ACVRL1*, OMIM #600376), JPHT (*SMAD4*, (OMIM ##175050), and HHT type 5 (*GDF2*, OMIM # 615506). Numbering is complicated because HHT types 3 and 4 had been assigned to genomic regions identified by linkage analyses, but there is now no evidence for *HHT3* [46], and reference to *HHT4* that was also assigned by linkage studies several decades ago is discouraged [46]. Whole-genome sequencing initiatives have yet to identify further HHT genes, although a large number of genes separately cause different vascular malformation syndromes [47].

From the earliest genetic studies, HHT phenotypes were seen to be highly variable between affected members of the same family. With the wider introduction of molecular diagnostics, as for other inherited conditions, the potential paucity of clinical features has become apparent [48]. Large prospectively-accrued series have identified environmental factors that contribute to more severe clinical manifestations once AVMs are present, particularly anaemia aggravating high-output states [49] and associated with earlier mortality [50,51]. Additionally, we have identified that in HHT, iron deficiency is associated with venous thromboemboli [52] and ischaemic strokes through pulmonary AVMs [53]; iron treatments can be associated with worsening nosebleeds in approximately 1 in 20 patients [54,55]; higher serum iron levels and intravenous iron treatments are independent risk factors for cerebral abscess due to pulmonary AVMs [56]; and, most recently, that unsupervised machine learning identifies relevant heterogeneity in blood indices [57]. Despite HHT clinical risks, recent European studies have shown that life expectancy is normal, or near-normal, in HHT [50,58,59,60]. This led to the discovery that HHT patients have lower rates of cancer [58,61], with better survival rates described if cancer does occur [62]. Separately, lower rates of heart attacks are described in one survey-based study [63].

Mechanisms for some elements of HHT vascular variability are now being elucidated. For AVMs, pulmonary and cerebral are more common in *ENG* patients [64,65,66,67,68], hepatic AVMs are more common in *ACVRL1* patients [64,65,66,67,68], and independent variants in *PTPN14* and *ADAM17* are associated with pulmonary AVMs [69,70]. There is no association between nosebleed severity and HHT causal genes [71,72], but bleeding severity was higher in one study of 104 patients with HHT when, by chance, deleterious variants in platelet and coagulation bleeding disorder genes were also present [72]. Cellular ‘second hit’ causes of phenotypic variability are a major focus of research [73]: low-level somatic loss of the second wildtype allele [74], HHT endothelial compensation mechanisms [47,75], cellular stress responses [47,75,76], and pharmacogenomic considerations [77] are all under active study.

**Figure 1:**
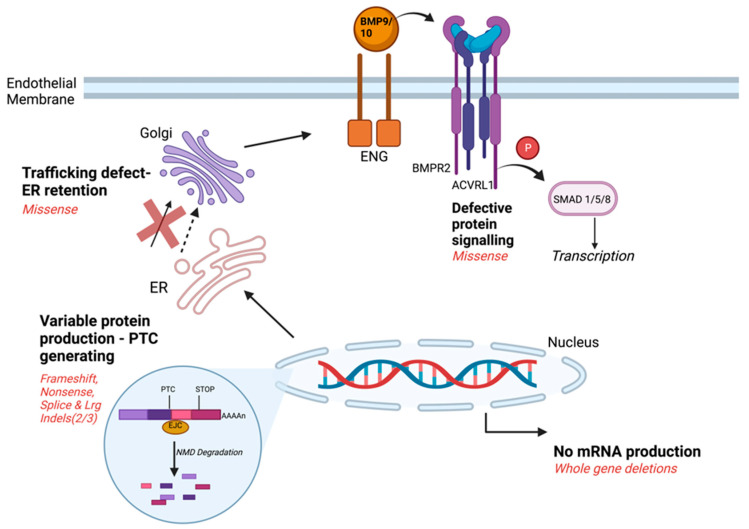
Relevant molecular considerations for the differing types of HHT causal pathogenic variants. A simple categorization of major conceptual classes of loss-of-function variants, distinguishing defective signalling variants from other classes that generate proteins that do reach the cell surface, or no protein: premature termination codons (PTCs) where aberrant protein generated varies [75], trafficking defect due to impaired protein folding [79,80,81], and whole gene deletions. Created using BioRender (Toronto, ON, Canada), Student Plan licence publication agreement number *FO25IXFDHY*, 24 June 2023).

Until now, relatively little attention has been directed to the precise molecular subtype of the HHT casual gene [65,75,76,78,79,80,81]. For *ACVRL1*, *ENG,* and *SMAD4*, multiple different loss-of-function variants are described, falling into expected molecular Sequencing Ontology (SO) terms [71,76,82]. In 2011, the number of reports that were publicly available suggested the number of times each variant was reported in different HHT families corresponded to first order decay kinetics suggesting a random origin [78]. However, as shown in Figure 1, potential consequences for the cell differ.

The current study originated in 2022, in order to examine phenotypic differences with differing types of HHT variants for reference [75] (Bernabeu-Herrero et al., under revision). Separately, in 2023, in order to emphasise how commonly patients with clinical HHT are not found to have causal mutations in disease genes to support reference [76], we formally audited a new series of HHT families where individuals had undergone clinical gene testing within the NHS Genomic Medicine Service, employing new levels of stringency towards pathogenicity assignment [83]. We noted a specific *ACVRL1* variant sequence type to be overrepresented in these clinical genetic tests and investigated further.

## 2. Methods

### 2.1. Study Populations

#### 2.1.1. Series 1: Hammersmith Hospital (Imperial) 2021–2023 Series

Clinical genetic diagnostics first explores if an affected family member is already known to have a molecular cause for their condition (when testing for that specific family variant is performed), and otherwise, proceeds to test a series of genes compatible with the phenotype. Within the UK, since 2019, the UK National Health Service (NHS) Genomic Test Directory [84] has defined which patients may have a clinical genetic test for HHT (under indication R186), and, where appropriate, following informed consent, a clinical NHS gene test can be requested, assuming individuals meet the indication criteria [48,84]. Upon the restart of face-to-face services following COVID-19 interruptions, as part of routine clinical care for individuals where HHT was suspected or definitely present, on the day of their HHT or AVM clinical assessment, they were offered a genetic test if this had not been performed previously in any known relative. In this index series, following patient consent, HHT genetic testing of family probands was requested by our clinical HHT service between 2 August 2021 and 6 March 2023. DNA was extracted, sequenced, and interpreted by and in a single NHS Genomic Medicine Service Specialist Laboratory that rigorously assigned molecular pathogenicity based on American College of Medical Genetics and Genomics and the Association for Molecular Pathology standards [83]. Case notes were subsequently reviewed with ethical approval from the Hammersmith and Queen Charlotte’s and Chelsea Research Ethics Committee (LREC 2000/5764).

#### 2.1.2. Series 2: Non-Overlapping Hammersmith Hospital (Imperial) 1992–1020 Series

As described elsewhere, between 1992 and 2020, HHT patients had genetic investigations through research protocols under ethical approvals (Scotland A MREC Ethics Committee (MREC 98/0/42 and 07/MRE00/19) or clinical genetic tests, including through the 100,000 Genomes Project (ethical approval by the Health Research Authority (HRA) Committee East England–Cambridge South (REC Ref 14/EE/1112)). Case notes were reviewed with ethical approval from the Hammersmith and Queen Charlotte’s and Chelsea Research Ethics Committee (LREC 2000/5764). Key findings in this cohort referring to individual clinical features corresponding to the respective genetic variants were published previously [57,71,72,85]. In the current study, we focused on the variants and the number of times each variant was reported. We ensured that variants identified in known or likely relatives were counted only once by using family trees of at least 3 antecedent generations, including the maiden names of affected women, and only counting the variant once if present in two individuals from a similar part of the country.

#### 2.1.3. Series 3: International HHT Mutation Database

In 1996 and 1997, the first Scientific Meetings of the HHT Foundation International Inc. were held in Edinburgh and Curaçao to share the evolving knowledge of clinical and genetic HHT. The first two genes for HHT had been identified in 1994 [26] and 1996 [27], and it was recognised that, with an increasing number of international groups performing ethically approved research studies, and clinical genetic testing likely to be introduced through clinical diagnostic laboratories, it would be valuable to have an international database. The Molecular Diagnostic Laboratory in Edinburgh, supported by the HHT Foundation International Inc., was selected and compiled the publicly reported variants that were being published in individual ethically approved research studies. As described a few years later [86], at its inception in May 2005, it contained 112 *ENG* and 83 *ACVRL1* variants, and by 2008, it contained over 600 variants in the two genes, with 539 believed to be pathogenic (disease-causing). The database was transferred to the ARUP Laboratories, University of Utah, which housed the website until 1 August 2023, when clinical variant data were shared with ClinVar [87] and other databases. The data reported in this study were downloaded in 2018, as reported elsewhere [71].

### 2.2. Data Analysis

We analysed each series separately for statistical rigor, to capture graphically, and to display inter-series consistency. While we were confident that there were no recent family overlaps between the two local series, we could not be certain that different members of particular families were being reported only once by the multiple laboratories worldwide. On the other hand, each international laboratory was ‘counted’ as contributing only a single report; thus, Series 3 was confounded in both directions. Recognising this, we nevertheless included as a comparator to extend beyond the UK base of Series 1 and 2.

Additionally, we approximated an aggregation between each different family report (Series 1 and 2), and each laboratories’ report (Series 3), as there were no known overlaps between the families in the two local datasets and no known overlap to the *ACVRL1* families reported by the HHT Mutation Database. Each variant was assigned to a row, and the number of reports in each database for each variant were entered. These values were then added to give an approximated ‘total’ number of reports.

Descriptive, comparative, and relationship statistics were generated using STATA version 15 (StataCorp, College Station, TX, USA) and GraphPad Prism 9.2.0 (GraphPad Software, San Diego, CA, USA). Two group comparisons were performed for continuous data via the Mann–Whitney test, and for categorical data, via the chi-squared (χ^2^) test. Graphical representations were performed using R and GraphPad Prism 9.2.0 (GraphPad Software, San Diego, CA, USA).

## 3. Results

### 3.1. HHT Variant Distribution Patterns

#### 3.1.1. Series 1

Between 2 August 2021 and 6 March 2023, 61 requests for NHS gene tests were submitted by our clinical HHT service on unrelated individuals with a definite, suspected, or possible clinical diagnosis of HHT, where no known relative had previously been genetically tested. In total, 25 gene-tested individuals met three Curaçao criteria (i.e., a definite clinical diagnosis of HHT), and 36 met fewer than three criteria (i.e., possible or suspected HHT). A total of 37 positive results were returned in addition to 24 negative results for *ACVRL1*, *ENG*, *SMAD4*, *GDF2*, *EPHB4,* and *RASA1*. For individuals receiving a positive gene test, 19/37 (51%) met three Curaçao criteria, compared to 6/24 (25%) of those receiving a negative gene test (χ^2^ *p* = 0.041). The 24 with a negative gene test were gene tested with varying degrees of clinical suspicion of HHT and were a disparate group, with 1–4 (mean 2) Curaçao Criteria, including 15 (62.5%) with pulmonary AVMs. Three (12.5%) had a family history of clinically-confirmed HHT, and a further nine (37.5%) had a family history of nosebleeds. This group was not considered further for the current manuscript.

The 37 individuals with a positive HHT gene test ranged in age from 19 to 75 (mean 38) years. Twenty-six (70.3%) were female, and nine (24.3%) were of non-European ethnicity. Again, they were gene tested with varying degrees of clinical suspicion of HHT. Individuals in this group had 1–4 (mean 2.5) Curaçao Criteria at the time the positive gene test was sent, including 21 (56.7%) with pulmonary AVMs. Only 4 (10.8%) had a formal clinical family history of HHT, but a further 26 (70.3%) had a family history of nosebleeds.

**Figure 2:**
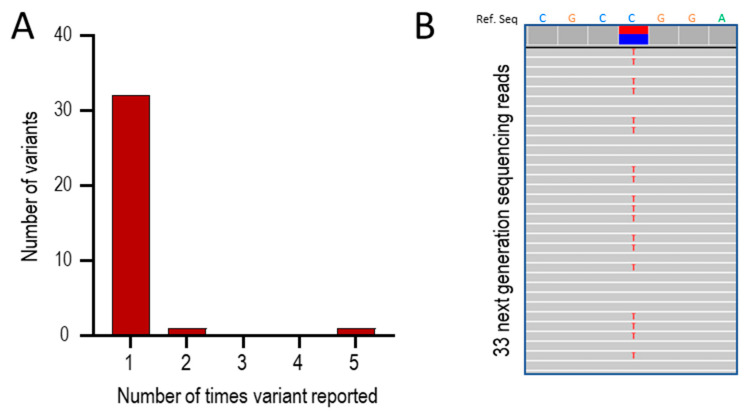
Series 1: Positive HHT gene tests between 2021 and 2023. (**A**) Overall number of times a variant was reported. (**B**) Sequence traces in one of the five patients with *ACVRL1* c.1231C>T (p.Arg411Trp). The upper bar indicates the reference sequence for 7 nucleotides spanning the variant, where the heterozygous call is highlighted by the blue/red horizontal bars. The 33 numbered rows show individual next-generation sequencing reads where variants are highlighted by letter. At Chr12:51,916,218,308, the genomic site corresponding to *ACVRL1* c.1231C, 308 (53%) of reads were the wild type C sequence (blue) and 275 (47%) were the variant sequence (red, indicated by an individual red ‘T’ on each sequence trace).

The 37 positive results represented 32 distinct pathogenic or likely pathogenic [83] variants in *ACVRL1*, *ENG,* and *SMAD4*. Thirty variants (94%) were present in only one individual/family, and one was identified in two individuals/families that were not knowingly related (Figure 2A). Strikingly, there were five separate reports in five different family probands of *ACVRL1* c.1231C>T (p.Arg411Trp; Figure 2A,B). This pathogenic missense variant substitutes tryptophan (Trp, W) for arginine (Arg, R) at codon 411 of the activin receptor-like kinase (ALK)1 protein encoded by *ACVRL1* [88]. The five families were not knowingly related, though all were of European (UK) origin.

#### 3.1.2. Series 2

**Figure 3:**
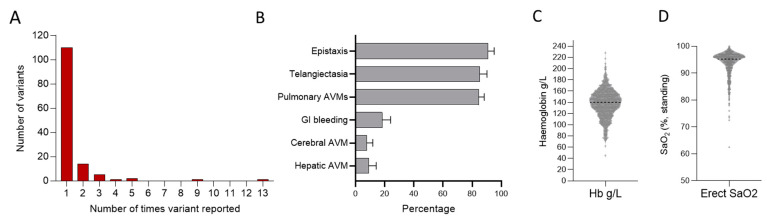
Series 2. Positive HHT gene tests in 501 patients with HHT reviewed between 1992 and 2020. (**A**) Overall number of times a variant was reported. (**B**) Major HHT manifestations across the population including nosebleeds (epistaxis), arteriovenous malformations (AVMs), and gastrointestinal (GI) bleeding. Note there is a strong referral bias due to a separate national pulmonary AVM referral service. Additionally, individuals referred with known or suspected HHT undergo routine screening for pulmonary AVMs, whereas imaging of the liver and brain for asymptomatic screens are not routinely performed. (**C**) First-recorded haemoglobin (Hb) and (**D**) first-recorded oxygen saturation (SaO_2_) in the erect posture.

In research and clinical gene tests on Hammersmith patients and their families between 1992 and 2020, 134 unique pathogenic variants were identified in *ACVRL1*, *ENG*, *SMAD4,* or *GDF2* (Figure 3A). These were identified in 501 patients, who, at presentation, had a mean age of 51 years, with 298 (60%) being female. As shown in Figure 3B, there was a pulmonary AVM bias in this series. The mean haemoglobin at first presentation was high normal (Figure 3C), driven by polycythaemia in patients with significant hypoxaemia due to pulmonary AVMs (Figure 3D).

In this series, 110 (82.1%) of the 134 unique disease-causal variants were identified in only one family, and 132/134 (98.5%) in five or fewer families (Figure 3A). However, two variants were reported in 9 and 13 different, not knowingly related families (Figure 3A). The *ACVRL1* c.1231C>T (p.Arg411Trp) variant was again an outlier, reported nine times. *ENG* c.277C>T (p.Arg93*) was reported 13 times, and is studied further elsewhere [75].

#### 3.1.3. HHT Mutation Database

**Figure 4:**
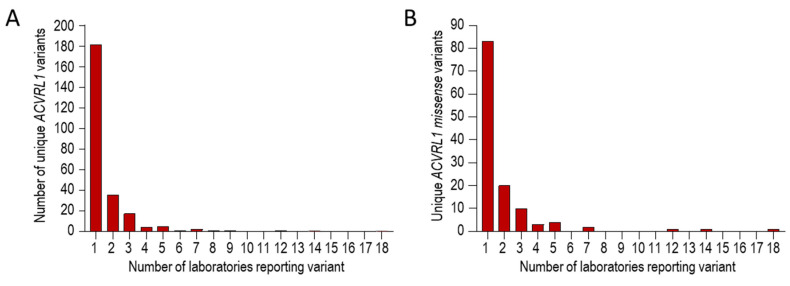
Series 3 *ACVRL1* pathogenic reports on the HHT Mutation Database, itemised by number of different international laboratories reporting the variant [41,79,89,90,91,92,93,94,95,96,97,98,99,100,101,102,103,104,105,106,107,108,109,110,111,112,113,114,115,116,117]. Tabulated text data were downloaded in 2018 as described in [71], and were categorized before generating these graphs. (**A**) Overall number of times any *ACVRL1* pathogenic variant was reported. (**B**) Overall number of times any missense *ACVRL1* pathogenic variant was reported.

To test if the *ACVRL1* c.1231C>T (p.Arg411Trp) predominance was also evident in non-UK laboratories, we examined pathogenic reports we had downloaded from the HHT Mutation Database as part of a separate study [71]. These reports had been generated by North American, European, African, and Asian genetic laboratories [41,79,89,90,91,92,93,94,95,96,97,98,99,100,101,102,103,104,105,106,107,108,109,110,111,112,113,114,115,116,117]. Within this database, there were 250 different reports for pathogenic *ACVRL1* variants. Of these, 181/250 (72.4%) were reported only once, and 247/250 (98.8%) fewer than ten times (Figure 4A). Focusing on the 125 *ACVRL1* missense variants, 83 (66.4%) were reported only once, and 122/125 (97.6%) fewer than eight times (Figure 4B).

Three variants were reported by more than 10 different laboratories. These were *ACVRL1* c.1231C>T (p.Arg411Trp), *ACVRL1* c.1232G>A (p.Arg411Gln), and *ACVRL1* c.1120C>T (p.Arg374Trp), reported by 12, 14, and 18 different laboratories, respectively. *ACVRL1* c.1121G>A (p.Arg374Gln) was the next most common variant (Table 1).

#### 3.1.4. Combined Analyses

**Figure 5:**
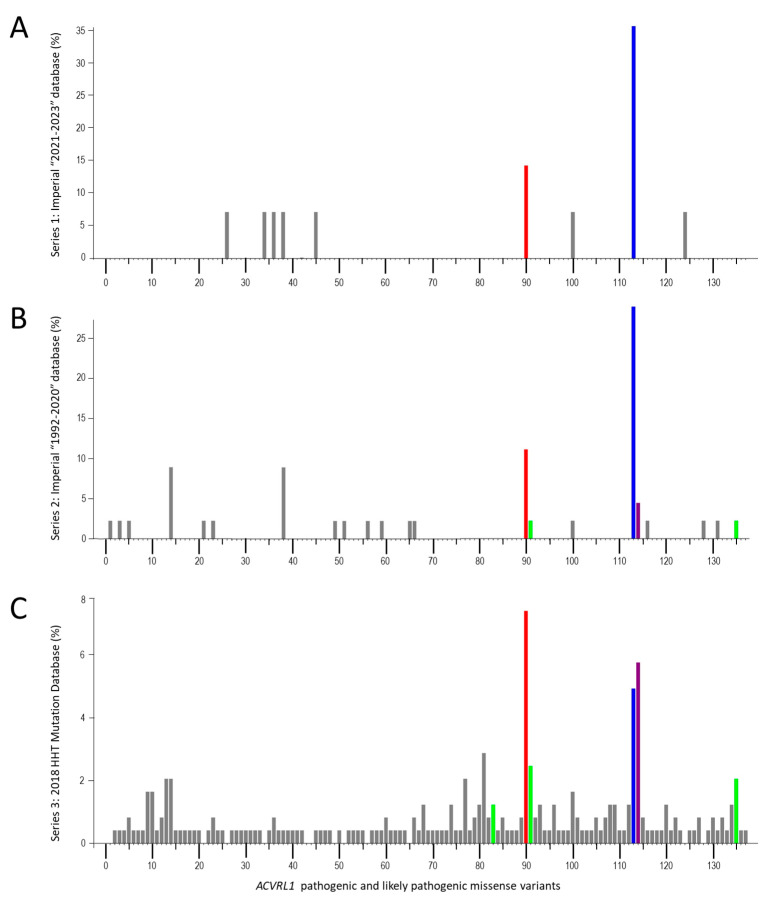
*ACVRL1* missense variants in three essentially independent series, plotted as percentage of total *ACVRL1* missense variants per series. Individual variants are numbered from 5′ to 3′ and detailed in Supplementary Table S1, annotated by NM_000020.3 [88]. (**A**) Series 1: Hammersmith/Imperial database, 2021–2023. (**B**) Series 2: Hammersmith/Imperial database, 1992–2020. (**C**) Series 3: HHT Mutation Database, 2018, as reported in Reference [71]. The three most common variants are highlighted in red (*ACVRL1* c.1120C>T, p.(Arg374Trp), blue (*ACVRL1* c.1231C>T, p.(Arg411Trp), and purple (*ACVRL1* c.1232G>A, p.(Arg411Gln). Also highlighted in green are the other cell surface-expressed, BMP9-binding but kinase-dead variants in the series (*ACVRL1* c.1039G>C, p.(Arg347Pro); *ACVRL1* c.1121G>A, p.(Arg374Trp); and *ACVRL1* c.1450C>T, p.(Arg484Trp)).

There were 14 reports of *ACVRL1* missense variants in Series 1, 45 in Series 2, and 243 in Series 3. These comprised 137 different pathogenic or likely pathogenic *ACVRL1* missense variants (Supplementary Table S1). In total, 89 (65%) of these disease-causal variants were reported only once, and 134/137 (98%) fewer than ten times. The three most frequently reported variants were *ACVRL1* c.1231C>T, p.(Arg411Trp [R411W]); *ACVRL1* c.1120C>T, p.(Arg374Trp [R374W]); and *ACVRL1* c.1232G>A, p.(Arg411Gln [R411Q]) (Figure 5).

### 3.2. HHT Variant Classification Patterns

As illustrated in Figure 1, HHT-causal, loss-of-function *ACVRL1* missense variants can result in no protein production, retention of misfolded proteins within the endoplasmic reticulum and intracellular degradation, or cell surface expression of inactive protein [79,80,81].

Notably, the three most frequently reported variants (R411W, R374W, and R411Q) across the three HHT data series each substitute an essential ALK1 kinase domain amino acid, are expressed at the cell surface and able to bind BMP9 [79], but are unable to generate a cellular BMP9 response [79]. The data series presented in Figure 5 included three other variants where encoded ALK1 proteins were shown by Ricard et al. [79] to be cell surface retained, able to bind BMP9, but failing to generate a BMP9 response: *ACVRL1* c.1121G>A, p.(Arg374Gln [R374Q]); *ACVRL1* c.1450C>T, p.(Arg484Gln [R484Q]); and *ACVRL1* c.1039G>C, p.(Ala347Pro [A347P]). These were, respectively, present/reported seven, six, and three times in the three HHT series (Figure 5).

**Figure 6:**
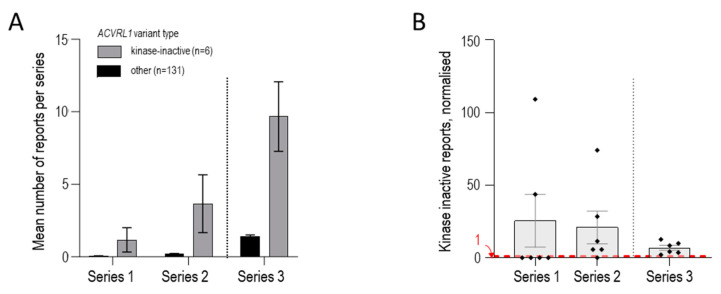
*ACVRL1* missense variants comparing the 6 encoding kinase-inactive, cell surface-expressed ALK1 protein compared to 131 other *ACVRL1* pathogenic/likely pathogenic missense variants reported in the 3 series as detailed in Supplementary Table S1. (**A**) Mean number of reports of individual variants per category (encoding cell surface-expressed, kinase-inactive protein [N = 6] or other missense variant [N = 131]) in each of the 3 series. Mean and standard error of mean reports per variant displayed. (**B**) Number of reports for each of the 6 kinase-inactive variants per series, normalised to the mean number of reports for the other *ACVRL1* variants in that series. Individual variant values, mean, and standard error of mean are displayed. For Series 1 and 2, kinase-inactive variants that were not identified have a value of zero (compare Figure 5). A red dotted line at 1 indicates where the expected value would be if, for a kinase-inactive variant present in that series, there was no difference from the other *ACVRL1* variants in that series.

We therefore compared the number of reports for the 6 known ‘cell surface-expressed, kinase-dead’ variants with the remaining 131 *ACVRL1*/ALK1 missense variants. In Series 1, the mean number of reports was 1.17 for the 6, compared to 0.046 for the 131 (*p* = 0.0038, Figure 6A). In Series 2 and 3, the mean numbers of reports per total *ACVRL1* missense variants were 3.67 and 9.67 for the 6 respectively, compared to 0.18 and 1.41 for the 131 (both *p*-values < 0.0001, Figure 6A). Comparisons of the number of reports for the 6 kinase-inactive variants normalised to the mean number of reports for the other 131 variants in each of the three series are presented in Figure 6B.

Across the three series, the fold increase ranged from 6.8 to 25.5 (mean, 8.9). In other words, loss-of-function, HHT-causal *ACVRL1*/ALK1 missense variants where proteins reach the cell surface but fail to signal, can be estimated to be ~9-fold more common in HHT than other missense *ACVRL1* variants causing HHT.

### 3.3. Non-Biological Considerations

We considered potential non-biological explanations for this distribution pattern. Chance was unlikely, with the *p*-values indicating that the finding would be expected less than one in 10,000 times, and potentially less than 1 in 2.63 × 10^11^ times if all databases were fully independent. The three data series spanned 4 decades of identification in more than 20 different laboratories (Table 1), meaning it was difficult to propose the findings resulted from ‘technical’ reasons. Furthermore, as the variants were identified in patients from populations from at least 14 countries across four continents (Table 1), it was difficult to propose a founder effect, even if that appeared attractive in Series 1. We therefore considered biological possibilities.

### 3.4. Mutational Hot Spot Examinations

A DNA-based hypothesis (‘mutational hotspot’ or other) could not be completely discounted—but there was no evidence in support on inspecting the University of California Santa Cruz (UCSC) Genome Browser [118]: *ACVRL1* exon 8 nucleotides encoding Arg374 (CGG) lie 4–6 nucleotides upstream to an alternate transcriptional start site (Met376); the third nucleotide has a high FANTOM5 cap analysis of gene expression (CAGE) score [119], but Met376 first and second nucleotides do not, and, in any case, none of those features is shared by the nucleotides encoding A347, R411, or R484 [118].

### 3.5. Selective Pressure Considerations

Exome Aggregation Consortium (ExAc) data show that loss-of-function rare variants causing autosomal dominant Mendelian diseases are depleted from the general population attributed to negative selective pressures [120]. We therefore tested the hypothesis that cell surface-expressed, kinase-dead ALK1 proteins may confer less of a disadvantage to the patient and be more likely to persist in the population. However, even before the introduction of specific HHT screening and treatment programmes, life expectancy in HHT was reasonably well preserved, with most patients reaching 70 years of age [51,60], and later studies show further improvements [58,59]. Thus, despite multiple deleterious HHT clinical features, a hypothesis based upon negative selection for HHT reasons was also difficult to support.

## 4. Discussion

We have shown, in three separate series of HHT-causal variants from 14 countries across four continents and from 4 decades of laboratory reports, that variants encoding ALK1 proteins that reach the cell surface but fail to signal were reported a mean of 6.8–25.5 (mean 8.9) times more frequently than the other *ACVRL1* missense variants in the same series. The excess of kinase-inactive, cell-surface expressed *ACVRL1*/ALK1 pathogenic missense variants has not been previously appreciated and could not be clearly explained by mutational hot spots or negative selection pressures.

In the wider medical literature, there are well-recognised examples where potentially ‘deleterious’ variants or mutations can result in individual health benefit, including Sickle Cell Trait (for malaria resistance) [121] and HIV resistance for people with a 32 bp deletion in *CCR5* [122], with further examples emerging [123]. We therefore considered if potential advantages might be conferred by *ACVRL1*/ALK1 variants where proteins reach the cell surface but fail to signal.

New laboratory data in early 2023 [124] provided a tantalising potential mechanism: separately from BMP signalling roles, ALK1 mediates low-density lipoprotein (LDL) transcytosis into arterial endothelium [125]. In 2023, genetic arterial-specific deletion of ALK1 and monoclonal antibody blockade of ALK1 were shown to block LDL transcytosis and reduce atherosclerosis in vitro and in LDL receptor knockout mice fed a high-fat diet [124]. It should be emphasised that the authors used specific experimental methods to avoid inducing HHT-like vascular malformations (using a novel antibody chosen specifically because it blocked LDL binding without affecting BMP signalling, and a *Bmx Cre* that they showed did not induce retinal or cerebral arteriovenous malformations) [125]. Nevertheless, human germline *ACVRL1* loss-of-function variants provide natural ALK1 depletion states that will impact arterial ALK1 required for LDL transcytosis.

**Figure 7:**
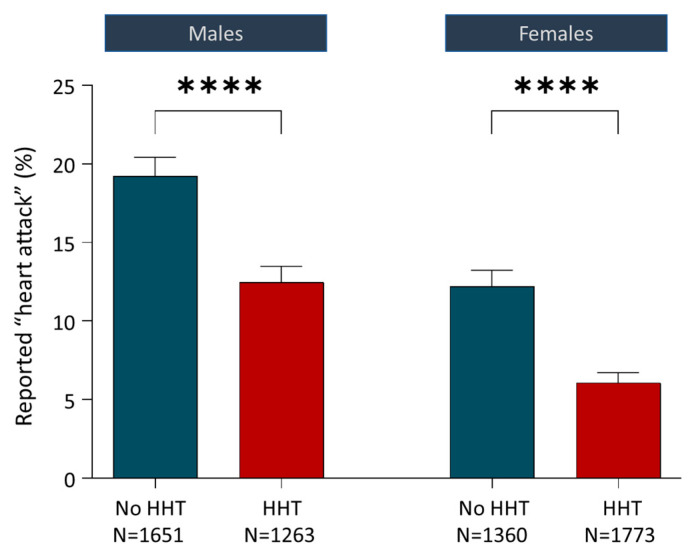
Relevant clinical considerations. Reported heart attacks (‘myocardial infarctions’) for 6047 individuals in hereditary haemorrhagic telangiectasia families comprising 2827 with HHT and 3011 without HHT. Data originally published in 2016 [63] were from 349 men and 670 women with HHT who also reported HHT transmission and cardiac events in 1978 parents and 3050 grandparents. Mean and standard error of the mean displayed; *p*-values calculated by Dunn’s test post Mann–Whitney (****, *p* < 0.0001). Reprinted/adapted with permission from Ref. [63]. Copyright year 2016, copyright owner’s name: Claire Shovlin.

Notably, clinical data in HHT support a hypothesis of reduced atherosclerosis. Reduced end-organ phenotypes were first reported in HHT by Alan Guttmacher in 1997, using a survey where HHT patients self-reported low rates of atherosclerosis and angina [126], concluding “If the suggestion of relatively low incidence of atherosclerosis in a primary disease of the vasculature persists after tabulation of remaining questionnaires, it deserves more careful analysis.” We also reported anecdotal data in 2009, based on the paucity of thrombolytic queries for HHT patients in a then 800-strong clinical service [127]. These observations [126,127] led to incorporation of unbiased cardiac histories in a wider ethically approved survey designed to capture multiple datasets from antecedent relatives of each respondent [63]. As shown in Figure 7, for 6047 individuals in HHT families (2827 with HHT and 3011 without HHT), whether categorised by all cases, or by males and females separately, the HHT group had fewer reported myocardial infarctions (heart attacks, all *p*-values < 0.0001) [63]. Given the potential HHT-specific complications that would confound any benefit from reduced atherosclerosis in HHT (e.g., higher cardiac outputs due to anaemia and systemic AVM-induced reductions in systemic vascular resistance; and paradoxical emboli through pulmonary AVMs that can occlude cerebral and coronary arteries directly), the HHT data shown in Figure 7 are remarkable and, until now, defied explanation.

Genotypes were not available for these families but may be estimated from prevalence patterns. In early 2023, more than 90% of the HHT causal variants on the *ACVRL1*, *ENG,* and *SMAD4* HHT Mutation Databases were in *ACVRL1* or *ENG*, with a reasonably equal split [71,85]. Importantly, ALK1 deficiency is not limited to HHT patients with *ACVRL1* ‘mutations’. Multiple studies using HHT patient-derived endothelial cells have shown that *ENG*^+/−^ cells also demonstrate reduced ALK1 RNA and protein, attributed to adaptive responses [75,96]. In other words, the predominant HHT genotypes (*ACVRL1^+/^*^−^ and *ENG^+/^*^−^) both represent an ALK-1-deficient state in which reduced rates of myocardial infarction were reported by patients in an unbiased survey.

This would explain pan-HHT protection but does not in itself provide a mechanism for a specific advantage from cell surface-expressed, non-signalling *ACVRL1*/ALK1 missense variants. However, a plausible mechanism can be proposed based on ALK1 protein interactions. As noted in the introduction, the ALK1 ligand BMP9, which binds ALK1 with high affinity [33,34], does so in dimeric form. Each ‘monomer’ in the BMP9 homodimer has two binding sites, described as the ‘wrist’ (the pre-helix loop and α1 helix) and the ‘knuckle’ (a region of two antiparallel β-sheets). In dimeric BMP9 confirmation, this enables the binding of four receptors in the signalling complex—initially two ALK1 and two endoglin molecules (with endoglin first capturing BMP9 before presentation to ALK1 [35]), and subsequently two ALK1 and two type II receptors enabling propagation of BMP9 signals into the cell. Simplistically, if 50% of cell surface ALK1 is missing due to a different (heterozygous) HHT-causal variant (see Figure 1), then cell surface expression will be exclusively from the residual wild-type allele, whereas if 50% of cell surface ALK1 is ‘kinase-dead’, dimerisation with wild type has the potential to impede wild-type ALK1 function in a dominant negative manner. In addition to proposing general scenarios in which such dominant-negative activity could enhance benefit (e.g., reducing conceptus loss rates in early pregnancy given ALK1′s placental expression [128]), the new discovery that ALK1 depletion reduces LDL transcytosis and atherosclerosis allows for the postulation of hypotheses applying to childhood, early adulthood, or later adult life based on enhanced athletic vigour and protection from arterial pathologies.

## 5. Conclusions

Across three multinational databases accrued over four decades, we have shown that loss-of-function, HHT-causal *ACVRL1*/ALK1 variants where proteins reach the cell surface but fail to signal are significantly more common in HHT than other HHT-causal missense *ACVRL1* variants. On the basis of current knowledge, the most realistic explanation appears to be cellular or evolutionary advantage. With recent evidence showing that ALK1 depletion reduces atherosclerosis in mice, and two ALK1 molecules in each BMP9-signalling complex, a testable, plausible explanation can be proposed based on dominant negative activity further reducing ALK1 function. We suggest this should be addressed in respect to LDL transcytosis, as this may accelerate development of new strategies to limit atherosclerosis for the general population. The findings also support a more granular description of HHT genotypes incorporating molecular subtypes.

## Figures and Tables

**Table 1:** Series 3. Laboratories reporting local detection of specific *ACVRL1* missense variants to the HHT Mutation Database from continents (Cont.) of North America (1); Europe (2); Africa (3); and Asia (4) [41,79,89,90,91,92,93,94,95,96,97,98,99,100,101,102,103,104,105,106,107,108,109,110,111,112,113,114,115,116,117]. Positive reports for *ACVRL1* c.1120C>T, p.(Arg374Trp [R374W]; *ACVRL1* c.1121C>T, p.(Arg374Gln [R374G]; *ACVRL1* c.1231C>T, p.(Arg411Trp [R411W]; and *ACVRL1* c.1232G>A, p.(Arg411Gln [R411Q], are shown in blue against each source.

	Wild Type	Arg374		Arg411		
	Variant	Trp	Gln	Gln	Trp	
Cont.	Countries	c.1120C>T	c.1121G>A	c.1232G>A	c.1231C>T	References
1	Canada					Abdalla et al., 2003 [89]
1	Canada					Abdalla et al., 2004 [90]
1	Canada					Abdalla et al., 2005 [91]
1	US (Utah)					Bayrak-Toydemir et al., 2004 [92]
1, 2	US and UK					Berg et al., 1997 [93]
2	Denmark					Brusgaard et al., 2004 [94]
3	Morocco, Senegal					Canzonieri et al., 2014 [95]
2	Spain					Fernandez-L et al., 2005 [96]
2	Spain					Fernandez-L et al., 2006 [97]
2	Spain					Fontalba et al., 2008 [98]
1	US (Utah)					Gedge et al., 2007 [99]
2	UK					Harrison et al., 2003 [100]
1, 2	US and UK					Johnson et al., 1996 [27]
2	Denmark					Kjeldsen et al., 2001 [101]
2	Germany					Kuehl et al., 2005 [102]
2	Italy					Lenato et al., 2006 [103]
2	France					Lesca et al., 2004 [104]
2	France					Lesca et al., 2006 [105]
4	China					Lin et al., 2001 [106]
2	Netherlands					Letteboer et al., 2005 [107]
1	US (Utah)					McDonald et al., 2011 [108]
3	South Africa					Mutize et al., 2020 [41]
1	Canada					Nishida et al., 2012 [109]
2	Italy					Olivieri et al., 2006 [110]
2	Italy					Olivieri et al., 2007 [111]
2	France					Ricard et al., 2010 [79]
2	Finland					Sankelo et al., 2008 [112]
2	Spain					Sanz-Rodriguez et al., 2004 [113]
2	Germany					Schulte et al., 2005 [114]
2	UK					Trembath et al., 2001 [115]
2	Germany					Wehner et al., 2006 [116]
4	China					Zhang et al., 2004 [117]
1	US					Reported ARUP Laboratories
2	UK					Submitted by Edinburgh

## Data Availability

Data supporting reported results can be found in the Data Supplement. Primary data from the 100,000 Genomes Project, which are held in a secure Research Environment, are available to registered users. Please see https://www.genomicsengland.co.uk/research/academic (accessed on 15 December 2023) for further information.

## Supplementary Materials

The following supporting information can be downloaded at https://www.mdpi.com/article/10.3390/jcm13010250/s1. A single file containing a Supplementary Table detailing all *ACVRL1* missense variants in the 3 series. Table S1, *ACVRL1* HHT-causal missense variants across 3 data series.
