# Supplementary material for: Pathogenic Variant Frequencies in Hereditary Haemorrhagic Telangiectasia Support Clinical Evidence of Protection from Myocardial Infarction
Source: J Clin Med. 2023 Dec 31;13(1):250. doi: 10.3390/jcm13010250 (PMC10779873; doi:10.3390/jcm13010250)
Supplement: Supplementary file 1 [file jcm-13-00250-s001.zip › jcm-2748090-supplementary.pdf]

Pathogenic Variant Frequencies in Hereditary Haemorrhagic Telangiectasia Support Clinical Evidence of Protection from Myocardial Infarction.

Kinshuk Jain, Sarah C. McCarley, Ghazel Mukhtar, Anna Ferlin, Andrew Fleming, Deborah J Morris-Rosendahl, Claire L Shovlin

Data Supplement

TABLE S1: *ACVRL1* HHT-causal missense variants across 3 data series

TABLE S1:

ACVRL1 **HHT-causal missense variants across 3 data series**

Missense	c.	p.	Series 3	Series 2	Series 1
1	c.100T>C	p.Cys43Arg		1	
2	c.101G>A	p.Cys34Tyr	1		
3	c.107G>A	p.Cys36Tyr	1	1	
4	c.121T>C	p.Cys41Arg	1		
5	c.140G>C	p.Arg47Pro	2	1	
6	c.142G>A	p.Gly48Arg	1		
7	c.143G>A	p.Gly48Glu	1		
8	c.148T>G	p.Try50Gly	1		
9	c.150G>T	p.Trp50Cys	4		
10	c.152G>A	p.Cys51Tyr	4		
11	c.154A>G	p.Thr52Ala	1		
12	c.197A>C	p.His66Pro	2		
13	c.199C>T	p.Arg67Trp	5		
14	c.200G>A	p.Arg67Gln	5	4	
15	c.202G>T	p.Gly68Cys	1		
16	c.205T>C	p.Cys69Arg	1		
17	c.206G>A	p.Cys69Tyr	1		
18	c.230G>A	p.Cys77Tyr	1		
19	c.231C>G	p.Cys77Trp	1		
20	c.235G>A	p.Gly79Arg	1		
21	c.236G>A,	p.Gly79Glu		1	
22	c.266G>A	p.Cys89Tyr	1		
23	c.269G>A	p.Cys90Tyr	2	1	
24	c.283T>C	p.Cys95Arg	1		
25	c.286A>G	p.Asn96Asp	1		
26	c.293A>C	p.(Asn98Thr)			1
27	c.293A>G	p.Asn98Ser	1		
28	c.88C>T	p.Pro30Ser	1		
29	c.95T>G	p.Val32Gly	1		
30	c.383C>A	p.Ala128Asp	1		
31	c.526G>T	p.Asp176Tyr	1		
32	c.536A>C	p.Asp179Ala	1		
33	c.586A>G	p.Arg196Gly	1		
34	c.589A>G	p.(Thr197Ala)			1
35	c.590C>T	p.Thr197Ile	1		...../
36	c.632G>A	p.Gly211Asp	2		1
37	c.632G>C	p.Gly211Ala	1		
38	c.643G>A	p.Glu215Lys	1		1
39	c.656G>A	p.Gly219Asp	1		
40	c.656G>A	p.Gly219His	1		
41	c.667G>C	p.Gly223Arg	1		
42	c.670G>A	p.Gly224Lys	1		
43	c.632G>A	p.Gly225Asp		4	
44	c.676G>A	p.(Val226Met)			1
45	c.686A>G	p.Lys229Arg	1		
46	c.686A>T	p.Lys229Met	1		
47	c.698C>T	p.Ser233Leu	1		
48	c.709C>A	p.Gln237Lys	1		
49	c.750G>C	p. Leu250Phe		1	
50	c.778A>C	p.Ile260Leu	1		
51	c.787G>A	p.Asp263Asn		1	
52	c.793A>C	p.Thr265Pro	1		
53	c.818T>C	p.Leu273Pro	1		
54	c.827T>C	p.Ile276Thr	1		
55	c.838C>A	p.His280Asp	1		
56	c.848G>T	p.Gly283Val		1	
57	c.851C>T	p.Ser284Phe	1		
58	c.853C>T	p.Leu285Phe	1		
59	c.866T>C	p.Leu289Pro	1	1	
60	c.875A>C	p.Gln292Pro	2		
61	c.890A>G	p.His297Arg	1		
62	c.905T>G	p.Leu302Arg	1		
63	c.913T>C	p.Ser305Pro	1		
64	c.916G>C	p.Ala306Pro	1		
65	c.920C>A	p.(Ala307Glu).		1	
66	c.925G>A	p.Gly309Ser	2	1	
67	c.925G>T	Gly309Cys	1		
68	c.940C>T	p.His314Tyr	3		
69	c.956G>A	p.Gly319Asp	1		
70	c.983A>C	P.His328Pro	1	...../	

…./

Missense	c.	p.	Series 3	Series 2	Series 1
71	c.983A>C	p.His328Pro	1		
72	c.986G>A	p.Arg329His	1		
73	c.988G>A	p.Asp330Asn	1		
74	c.988G>T	p.Asp330Tyr	3		
75	c.992T>C	p.Phe331Ser	1		
76	c.997A>G	p.Ser333Gly	1		
77	c.998G>T	p.Ser333Ile	5		
78	c.1010T>C	p.Leu337Pro	1		
79	c.1023C>G	p.Asn341Lys	2		
80	c.1030T>C	p.Cys344Arg	3		
81	c.1031G>A	p.Cys344Tyr	7		
82	c.1031G>T	p.Cys344Phe	2		
83	c.1039G>C	p.Ala347Pro	3		
84	c.1048G>A	p.Gly350Ser	1		
85	c.1048G>C	p.Gly350Arg	2		
86	c.1054G>C	p.Ala352Pro	1		
87	c.1055C>A	p.Ala352Asp	1		
88	c.1061T>A	p.Met354Lys	1		
89	c.1115C>T	p.Thr372Ile	2		
90	c.1120C>T	p.Arg374Trp	18	5	2
91	c.1121G>A	p.Arg374Gln	6	1	
92	c.1123T>C	p.Tyr375His	2		
93	c.1126A>G	p.Met376Val	3		
94	c.1127T>A	p.Met376Lys	1		
95	c.1127T>C	p.Met376Thr	1		
96	c.1127T>G	p.Met376Arg	3		
97	c.1129G>A	p.Ala377Thr	1		
98	c.1133C>A	p.Pro378His	1		
99	c.1133C>T	p.Pro378Leu	1		
100	c.1135G>A	p.Glu379Lys	4	1	1
101	c.1139T>G	p.Val380Gly	2		
102	c.1157G>A	p.Arg386His	1		
103	c.1189G>A	p.Asp397Asn	1		
104	c.1190A>G	p.Asp397Gly	1		
105	c.1193T>A	p.Ile398Asp	2		
106	c.1196G>C	p.Trp399Ser	1	…./	

…./

Missense	c.	p.	Series 3	Series 2	Series 1
107	c.1199C>A	p.Ala400Glu	2		
108	c.1204G>A	p.Gly402Ser	3		
109	c.1208T>C	p.Leu403Pro	3		
110	c.1218G>C	p.Trp406Cys	1		
111	c.1218G>T	p.Trp406Cys	1		
112	c.1221G>T	p.Glu407Asp	3		
113	c.1231C>T	p.Arg411Trp	12	13	5
114	c.1232G>A	p.Arg411Gln	14	2	
115	c.1232G>C	p.Arg411Pro	2		
116	c.1270C>A	p.Pro424Thr	1	1	
117	c.1270C>T	p.Pro424Ser	1		
118	c.1271C>T	p.Pro424Leu	1		
119	c.1273T>G	p.Phe425Val	1		
120	c.1275C>G	p.Phe425Leu	3		
121	c.1276A>G	p.Tyr426Cys	1		
122	c.1280A>T	p.Asp427Val	2		
123	c.1297C>T	p.Pro433Ser	1		
124	c.1298C>A	p.Pro433His			1
125	c.1298C>G	p.Pro433Arg	1		
126	c.1313T>C	p.Met438Thr	1		
127	c.1321G>A	p.Val441Met	2		
128	c.1325T>C	p.Val442Ala		1	
129	c.1346C>T	p.Pro449Leu	1		
130	c.1355C>T	p.Pro452Leu	2		
131	c1436G>A	pArg479Gln	1	1	
132	c.1436G>A	p.Arg479Glu	2		
133	c.1436G>T	p.Arg479Leu	1		
134	c.1445C>T	p.Ala482Val	3		
135	c.1450C>T	p.Arg484Trp	5	1	
136	c.1460A>C	p.Lys487Thr	1		
137	c.1475T>A	p.Ile492Asn	1		

Legend: Number of separate reports of stated pathogenic or likely pathogenic *ACVRL1* missense substitutions (GenBank RefSeq NM_000020.3) in three essentially independent series. Series 1: Imperial database from new family gene test results from 2021-2023; Series 2: Imperial database 1992-2020; Series 3: HHT Mutation Database as downloaded 2018 (for full description see main text). Data are plotted for each variant as percentage of total missense variants per database in main manuscript Figure 5, where numbers correspond to the numbers in this Table.
